# Gender differences in knee kinematics during weight-bearing knee flexion for patients with arthrofibrosis after anterior cruciate ligament reconstruction

**DOI:** 10.1186/s13018-021-02729-3

**Published:** 2021-09-26

**Authors:** Ling Zhang, Shuai Fan, Jiling Ye, Xin Jiang, Bin Cai

**Affiliations:** Department of Rehabilitation Medicine, Ninth People’s Hospital, Shanghai Jiao Tong University School of Medicine, 500th. Quxi Road, Shanghai, 200010 China

**Keywords:** Loss of motion, Gender difference, Kinematics, Patellar tracking, Fluoroscopy

## Abstract

**Background:**

Knowledge of tibiofemoral and patellofemoral joint kinematics is important for understanding gender-related dimorphism in developing knee arthrofibrosis and advancement of related treatments. The objective of our study was to investigate gender differences existing in tibiofemoral kinematics and patellar tracking in patients with arthrofibrosis after anterior cruciate ligament (ACL) reconstruction during weight-bearing knee flexion.

**Methods:**

The tibiofemoral and patellofemoral joint kinematics were measured in 30 patients (15 male and 15 female) with arthrofibrosis after ACL reconstruction during a lunge task, using computed tomography and dual fluoroscopic imaging system. These data were analyzed for gender differences.

**Results:**

The range of tibial rotation, patellar inferior shift, tilt, and flexion were significantly decreased in the affected knee compared to the contralateral knee from 15° to 75° of knee flexion (*P* ≤ 0.04). Statistically significant difference was detected for medial tibial translation between male and female patients at 60° (*P* = 0.04) and 75° of knee flexion (*P* = 0.02). The tibial rotation was significantly decreased at 60° (*P* = 0.03) and 75° of knee flexion (*P* < 0.01) in females. The inferior patellar shift in females was significantly lower than that in males at 15° (*P* = 0.04) and 30° of knee flexion (*P* = 0.01). The patellar tilt was significantly lower at 60° (*P* = 0.02) and 75° of knee flexion (*P* < 0.01) in females compared to males.

**Conclusions:**

The results indicated a significant effect of gender on knee kinematics in patients with arthrofibrosis after ACL reconstruction during weight-bearing knee flexion. These gender differences in tibiofemoral kinematics and patellar tracking may warrant further investigations to determine implications for making gender-specific surgical treatments and rehabilitation programs.

## Highlights

1. We investigated tibiofemoral kinematics and patellar tracking in patients with arthrofibrosis after ACL reconstruction.
2. We compared the in vivo 6 DOF tibiofemoral and patellofemoral kinematics during weight-bearing knee flexion.
3. A significant gender difference was found for knee kinematics in patients with arthrofibrosis after ACL reconstruction during weight-bearing knee flexion.
4. This knowledge is essential to understand the mechanisms underlying gender-related differences in developing knee arthrofibrosis after ACL reconstruction.
5. These results might help to make gender-specific treatment programs of knee arthrofibrosis.

## Background

Despite improvements in surgical techniques and rehabilitation programs, arthrofibrosis remains a common and devastating complication after anterior cruciate ligament (ACL) reconstruction, accounting for 2–5% of cases [1, 2]. Arthrofibrosis impairs gait and athleticism, and it has been suggested to increase the risk of developing knee osteoarthritis (OA) in the long term [3, 4]. Although management of knee arthrofibrosis has improved a lot in the past two decades and provided successful clinical outcomes, conservative and surgical modalities cannot totally restore normal function of the knee joint [4–6]. Mayr et al. [3] reported that long-term range of motion (ROM) improvement can be achieved by arthroscopic arthrolysis; however, patients complain of persistent symptoms, including decreased patellar mobility, anterior knee pain, and quadriceps weakness even after the intervention.

It has been reported that females have a 2.5-fold higher likelihood of developing arthrofibrosis after ACL reconstruction as compared to males [6–9]. Moreover, with arthrofibrosis after ACL reconstruction, female patients generally report worse outcomes scores than males on the self-reported knee function [3]. Early-onset knee OA following arthrofibrosis after ACL reconstruction has been reported in a growing number of studies [3, 5]. Females are also at a higher risk of developing knee OA in the long term, and it was suggested that the cartilage of females may be more vulnerable than that of males [3].

To date, the mechanism behind the development of arthrofibrosis following ACL reconstruction and the gender-specific difference of arthrofibrosis prevalence remain to be determined. Mikula et al. [10] suggested that one potential mechanism may be changes in knee biomechanics. In their in vitro study, which has quantified the six degrees-of-freedom (6 DOF) patellofemoral kinematics in the arthrofibrotic knee, they reported that abnormal positioning of the patella could result in changed patellofemoral contact biomechanics, which is associated with anterior knee pain, loss of motion, and extensor lag [10]. An in vivo study conducted by Zhang et al. [11] found that patients with arthrofibrosis after ACL reconstruction presented decreased patellar mobility in the arthrofibrotic knee compared with the contralateral knee. These suggestions that knee kinematics may be related to the mechanisms of loss of motion are interesting and potentially significant and also highlight the limited knowledge regarding the knee kinematics in patients with arthrofibrosis after ACL reconstruction.

Some authors suggest that gender-specific differences in knee kinematics during functional activities could influence knee injuries and the development of knee OA [12, 13]. Webster et al. [12] suggested that higher knee adduction moment seen in females compared with males may lead to an increased risk of developing OA in ACL-reconstructed females. Asaeda et al. [13] reported that more tibial external rotation seen in females compared with males may indicate a delay in the recovery of knee kinematics in ACL-reconstructed females, suggesting an increased risk of secondary OA after ACL reconstruction. Because gender-specific differences in knee kinematics have been identified after ACL reconstruction [12, 13], it could be speculated that gender specifics in knee kinematics could explain partly the gender difference in developing knee arthrofibrosis. Previous studies that have investigated risk factors of knee arthrofibrosis have not considered these gender-specific differences in their evaluation. So far, no in vivo study has been performed to investigate gender specifics in tibiofemoral and patellofemoral kinematics during a lunge task in patient with arthrofibrosis after ACL reconstruction.

Therefore, the purpose of our study was to compare the in vivo 6 DOF tibiofemoral and patellofemoral kinematics during weight-bearing knee flexion between males and females in patients with arthrofibrosis after ACL reconstruction, using three-dimensional (3D) computed tomography (CT) image-based models of the knee and a dual fluoroscopic imaging system (DFIS). We hypothesized that there would be a gender difference in the 6 DOF tibiofemoral and patellofemoral kinematics during weight-bearing condition in patients with arthrofibrosis after ACL reconstruction. This knowledge is essential to understand the mechanisms underlying gender-related differences in developing knee arthrofibrosis after ACL reconstruction. Additionally, this will help determine if there exists a justification for gender-specific treatment programs of knee arthrofibrosis.

## Materials and methods

### Participants

Thirty patients with arthrofibrosis after ACL reconstruction (15 females and 15 males) were included in this study. Arthrofibrosis of the knee is defined as a restricted ROM in extension and/or flexion that is attributable to soft tissue fibrosis that was present postoperatively, according to international consensus on the definition of fibrosis of the knee joint [14]. To determine knee arthrofibrosis, the cut-off angle was defined as for knee flexion range less than 100° and knee extension restriction greater than 5° [14]. Additionally, inclusion and exclusion criteria were as follows:

Inclusion criteria

1. within 6 w–24 w after unilateral ACL reconstruction;
2. knee flexion range < 100° or knee extension restriction > 5°;
3. have no previous surgery or other injuries to the affected knee.

Exclusion criteria

1. with malpositioned ligament reconstruction;
2. have injury to the hip joint or ankle joint for both limbs;
3. are currently undertaking physical therapy or other management practices, including conservative and surgical treatments.
4. have neuromuscular disorder of the lower extremity that affects knee function.

All patients underwent arthroscopic reconstruction of the double-bundle ACL with a hamstring tendon autograft. There was no significant difference for mean age and body mass index (BMI) between female and male patients (Table 1). However, male patients were taller (178.3 ± 9.2 cm vs 163.3 ± 6.2 cm, *P* = 0.01) and weighed more than female patents (75.2 ± 9.8 kg vs 58.2 ± 7.8 kg, *P* < 0.001). There was no significant difference for the International Knee Documentation Committee score (41.9 ± 5.6 vs 44.1 ± 6.8, *P* = 0.10) of the females and males. All patients underwent laboratory kinematic evaluation 1–2 days after recruitment. This study was approved by the University Review Board (SH9H-2019-T220-1) and registered with Chinese Clinical Trial Registry (ChiCTR1900025977). All patients provided their written informed consent prior to collecting data.

**Table 1:** Patient characteristics

		Participants		*P*-value
		Female (*n* = 15)	Male (*n* = 15)	
Age, y		33.8 ± 4.2	36.2 ± 6.3	0.88
Height, cm		163.3 ± 6.2	178.3 ± 9.2	0.01
Weight, kg		58.2 ± 7.8	75.2 ± 9.8	< 0.001
Body mass index, kg/m^2^		21.8 ± 4.2	23.7 ± 5.3	0.38
IKDC score		41.9 ± 5.6	44.1 ± 6.8	0.10

Values are presented as mean ± SD

ACL, anterior cruciate ligament; IKDC, International Knee Documentation Committee

### Procedures

#### Data collection

**Fig. 1:**
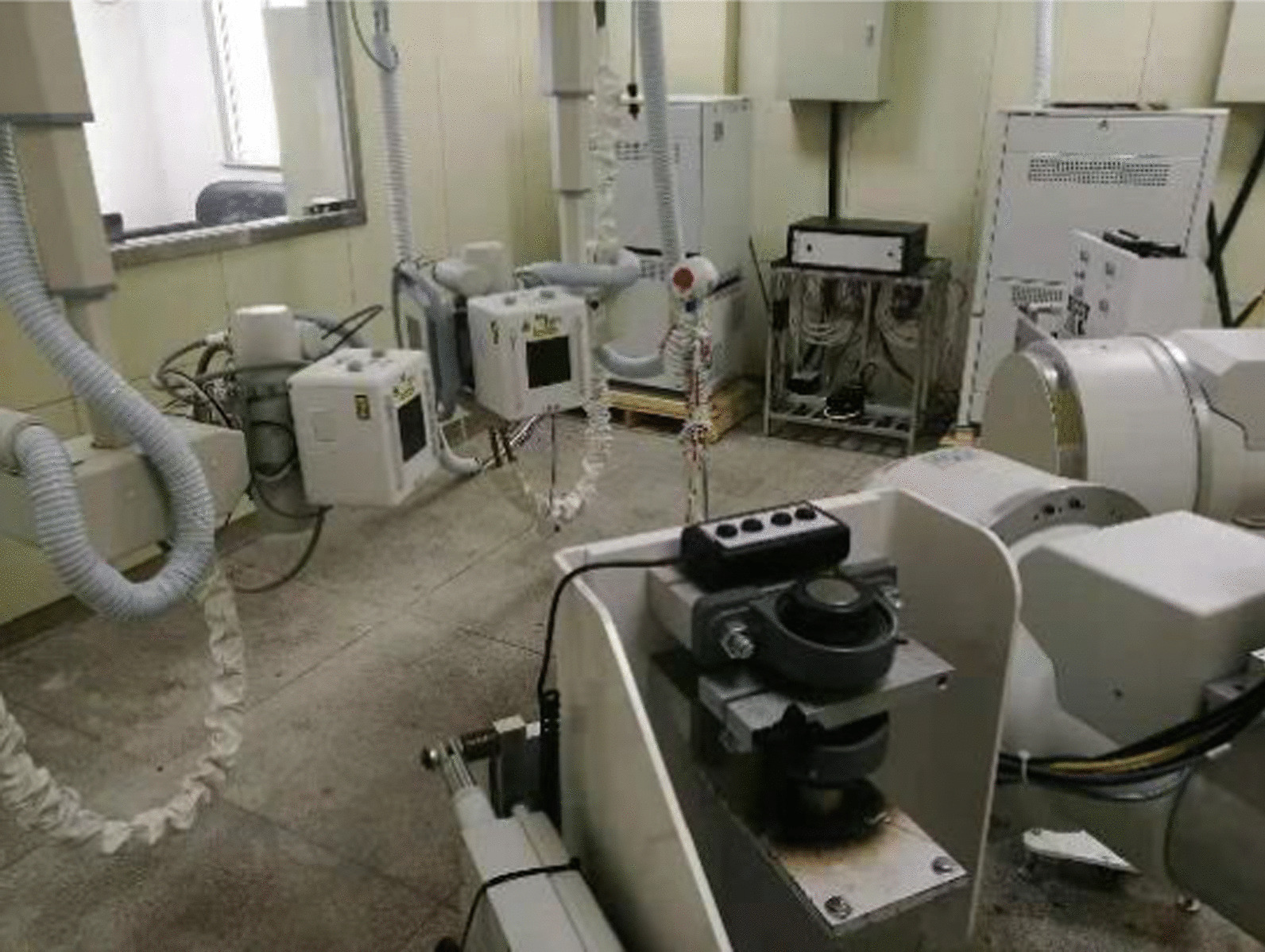
Experimental set-up

CT and dual fluoroscopic system were used to collect 3D kinematic data of both knees during a lunge task, which has been described in detail previously [11]. High repeatability and accuracy of this method have been reported previously [15]. CT scans (General Electric Medical Systems, Milwaukee, WI, USA) of each knee joint were captured in a 30 cm display field of view in the knee extension position (thickness 1 mm; resolution of 512 × 512 pixels). The CT data were imported into a solid modeling software (3D Slicer, www.slicer.org) to create the surface models of the femur, tibia, and patella. Then, all patients performed a single-leg lunge task (from the extended knee position to maximum flexion) in the view filed of a dual fluoroscopic system (65 kvp, 50 mA, and an average dose rate of 0.08 mSv/100 frame). The laboratory set up is shown in Fig. 1. Dynamic fluoroscopy images were captured for 10 s at 100 Hz throughout the whole lunge motion. Next, the fluoroscopic images were imported into custom MATLAB (R2018a; MathWorks, Inc., Natick, MA USA) and placed based on projection geometry of the fluoroscopes during the actual test [16, 17]. Afterwards, the CT-based 3D knee model was imported into this software and manipulated in 6 DOF until the projections of the knee model matched the outlines of the fluoroscopy images. Finally, when the projections of the knee models best matched the outlines of fluoroscopic images, the positions of the 3D models reproduced the in vivo 3D positions of the knee at each flexion angle [17].

#### Coordinate systems and description of knee motion

The coordinate systems of the femur, patella, and tibia were manually created to describe the motion of the tibiofemoral and patellofemoral joint, as previously described [16]. The femoral long axis was along the femoral shaft. The medial–lateral (ML) axis was along the transepicondylar axis (TEA), with the knee center at the midpoint of the TEA. The anterior–posterior (AP) axis was perpendicular to the other two axes. The tibial long axis was along the posterior wall of the tibial shaft. The ML axis was defined as a line connecting the center of the two circles fitting to the lateral and medial tibial plateau surfaces. The AP axis was perpendicular to the other two axes. For the patellar coordinate system, a cuboid was created to fit around the patella touching the contours of the patella in the AP, superior-inferior (SI), and ML directions. The geometric center of the cuboid was defined as the origin of the patella. The long axis of the patella was the line along the SI direction. The ML axis was the line connecting the medial and lateral ridges of the patella. The AP axis was perpendicular to the other axes.

The tibiofemoral kinematics were defined as the motion of the tibia relative to the femur, and the patellofemoral kinematics as the motion of the patella relative to the femur. The tibiofemoral rotation included flexion/extension, valgus/varus and internal/external rotation. The angle between the long axes of the femur and tibia in the sagittal plane was defined as the knee flexion. Tibial translations were defined as the translations of the tibial coordinate system along the AP, SI, and ML axes relative to the knee center. Patellar flexion was the rotation of the long axis of the patella around the TEA of the femur. Patellar tilt was considered as the rotation of the patella about its long axis. Patellar rotation was the rotation of the patella about its AP axis. Patellar ML shift referred to the medial or lateral translation of the center of the patella along the TEA of the femur. Patellar SI and AP shifts were the translations of the patellar center along the SI and AP axes relative to the knee center.

### Statistical analysis

All included patients had flexion and extension restrictions in the arthrofibrotic knee. To facilitate consistent comparisons between different patients and limbs, in vivo 3D tibiofemoral and patellofemoral motions were assessed with the knee flexion changing from 15° to 75° of knee flexion (defined as the angle between the long axes of the femur and tibia in the sagittal plane).

Continuous variables were calculated using means and standard deviations. A two-way analysis of variance (ANOVA) was used to determine main effects and interactions of gender and group on the range of tibial and patellar motion: two levels of gender (male and female) × two groups (the affected knee and the contralateral knee). Simple main effects were evaluated using a Bonferroni post hoc analysis after identification of significant interactions. To evaluate gender-specific differences at different knee flexion angles in the arthrofibrotic knee, a two-way ANOVA was used: two levels of gender (male and female) × five knee flexion angles of measurement (15°, 30°, 45°, 60°, and 75° of knee flexion). Again, with identified interactions and main effects, simple main effects were evaluated using a Bonferroni post hoc analysis. All analyses were performed using SPSS Statistics (IBM SPSS Statistics version 25.0, Armonk, NY, USA). The level of statistical significance was set at *P* < 0.05.

## Results

### Tibiofemoral and patellofemoral kinematics comparing for the contralateral knee

The range of tibial rotation in the affected knee was significantly lower than in the contralateral knee from 15° to 75° of knee flexion (*P* = 0.04, Table 2). Moreover, the range of tibial lateral translation, internal rotation, and valgus were lower in females than in males (*P* ≤ 0.03, Table 2). The range of patellar inferior shift, lateral tilt, and flexion were significantly decreased in the affected knee compared to the contralateral knee from 15° to 75° of knee flexion (*P* ≤ 0.04, Table 2). The range of patellar inferior shift and lateral tilt in males were significantly higher than in females (*P* ≤ 0.03, Table 2).

**Table 2:** Range of tibial and patellar motion

	Affected limb-M		Affected limb-F	Contralateral limb-M		Contralateral limb-F		Gender effect (*P*)	Group effect (*P*)	Gender × group interaction (*P*)
*Tibial motion*										
posterior translation, mm	8.9 ± 4.8		7.3 ± 4.1	7.6 ± 4.6		5.9 ± 3.8		0.13	0.87	0.58
inferior translation, mm	3.5 ± 1.8		2.9 ± 2.1	1.1 ± 1.8		1.9 ± 2.1		0.25	0.18	0.71
lateral translation, mm	1.5 ± 1.4		0.7 ± 1.5	2.2 ± 2.4		1.0 ± 2.5		**0.03**	0.38	0.40
valgus, °	4.0 ± 2.6		1.8 ± 2.5	4.6 ± 2.4		2.6 ± 2.1		**0.02**	0.21	0.14
Internal rotation, °	7.6 ± 4.3		3.9 ± 2.6	8.8 ± 4.5		5.6 ± 2.5		** < 0.01**	**0.04**	**0.01**
*Patellar motion*										
posterior shift, mm	21.9 ± 7.1		19.9 ± 6.8	25.3 ± 5.9		23.7 ± 6.8		0.41	0.75	0.55
inferior shift, mm	27.0 ± 5.1		21.6 ± 5.4	31.1 ± 4.7		26.9 ± 4.5		**0.01**	**0.02**	0.09
lateral shift, mm	3.7 ± 2.0		2.1 ± 1.8	3.5 ± 1.6		1.9 ± 1.8		0.26	0.42	0.30
flexion, °	34.4 ± 8.8		29.9 ± 7.8	45.4 ± 12.3		41.9 ± 9.8		0.43	**0.04**	0.24
Lateral tilt,°	0.6 ± 1.6		0.3 ± 0.8	1.5 ± 2.4		1.9 ± 2.8		**0.03**	** < 0.01**	**0.04**
Internal rotation, °	1.9 ± 1.7		1.5 ± 2.8	2.2 ± 2.3		1.9 ± 1.8		0.32	0.56	0.89

Bold values indicate statistically significant difference (*p* < 0.05)

Data are presented as mean ± SD

### Gender differences of knee kinematics in the arthrofibrotic knee

**Fig. 2:**
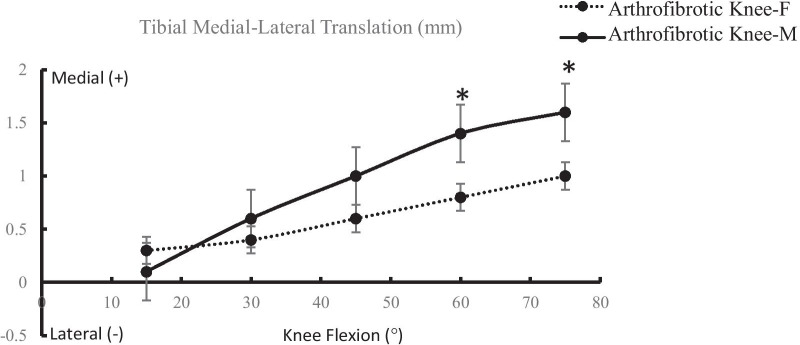
Medial tibial translation in the arthrofibrotic knee of males/females (*P < 0.05)

**Fig. 3:**
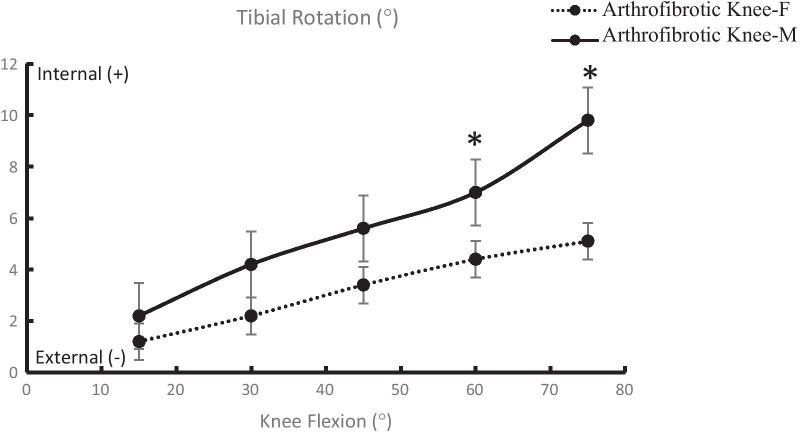
Tibial rotation in the arthrofibrotic knee of males/females (*P < 0.05)

The medial tibial translation increased from 15° to 75° of knee flexion in the arthrofibrotic knee. Statistically significant difference was detected for medial tibial translation between male and female patients at 60° (*P* = 0.04) and 75° of knee flexion (*P* = 0.02, Fig. 2). The arthrofibrotic knee demonstrated an internal tibial rotation throughout the knee flexion range. The tibial rotation was significantly decreased at 60° (*P* = 0.03) and 75° of knee flexion (*P* < 0.01) in females than in males (Fig. 3).

**Fig. 4:**
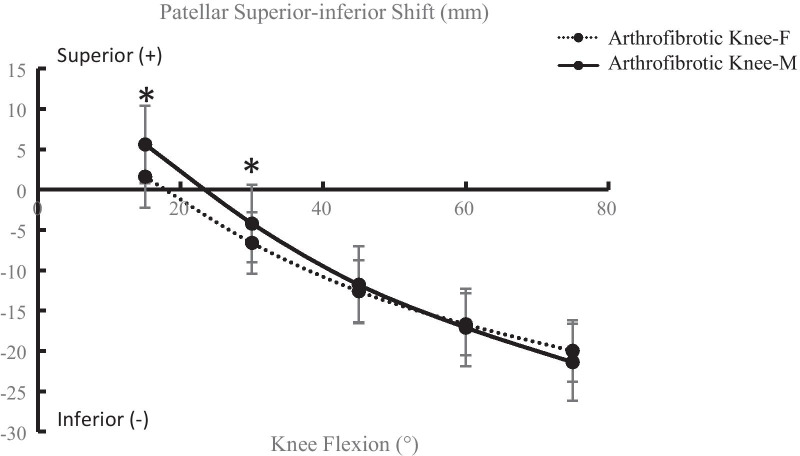
Inferior patellar shift in the arthrofibrotic knee of males/females (*P < 0.05)

**Fig. 5:**
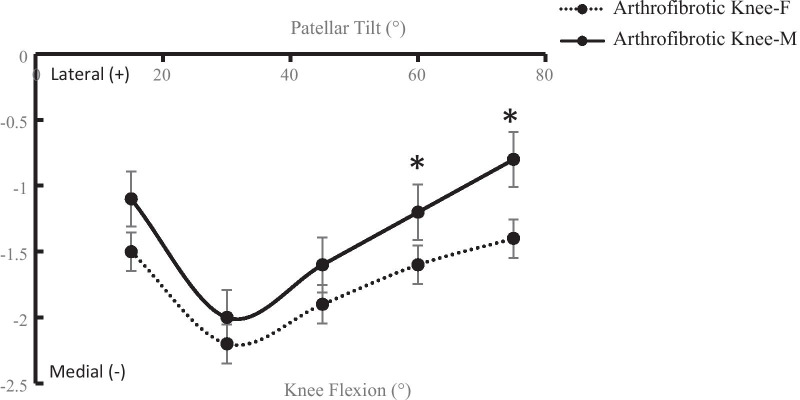
Patellar tilt in the arthrofibrotic knee of males/females (*P < 0.05)

During the lunge task, the patella consistently shifted inferiorly in the arthrofibrotic knee throughout the knee flexion range. The inferior patellar shift in females was significantly lower than that in males at 15° (*P* = 0.04) and 30° of knee flexion (*P* = 0.01) (Fig. 4). The patella tilted medially from 15° to 30° of knee flexion and then tilted laterally from 30° to 75°of knee flexion. The patellar tilt was significantly decreased at 60° (*P* = 0.02) and 75° of knee flexion (*P* < 0.01) in females compared to males (Fig. 5).

## Discussion

The present study investigated 6 DOF tibiofemoral and patellofemoral kinematics in patients with arthrofibrosis after ACL reconstruction, which was the first study to compare knee kinematics in male and female patients. Our study provides two important findings with regard to changes in tibiofemoral and patellofemoral kinematics in the arthrofibrotic knee. First, arthrofibrosis of the knee decreased tibial rotation and patellar mobility during dynamic knee flexion compared to the contralateral knee. Second, there existed gender differences in tibiofemoral kinematics and patellar tracking although many of the measured parameters were similar between genders.

Our study has showed that arthrofibrosis of the knee decreased the range of patellar inferior shift and flexion during 15°–75° of knee flexion. Mikula et al. [10] reported that arthrofibrosis in the suprapatellar pouch decreased patellar inferior shift and flexion. In the arthrofibrotic knee, fibrosis always involves the patellofemoral compartment, occurring in the infrapatellar fat pad, the suprapatellar pouch, and the anterior interval, which may restrict the patella to shift inferiorly during dynamic knee flexion [18]. Mauro et al. [19] reported that arthrofibrosis in the patellofemoral compartment may lead to patellar flexion loss. It is understandable because patellofemoral adhesions resulted in a decrease in patellar inferior shift, suggesting that the patella does not effectively engage the trochlea during dynamic knee flexion. Furthermore, the patella tilted significantly less laterally from 15° to 75° knee flexion in the arthrofibrotic knee compared to the contralateral knee. Contracture of the medial retinaculum of the knee may decrease lateral tilt of the patella [11]. Arthrofibrosis after ACL reconstruction also caused a decrease in the internal tibial rotation. It might be explained by the effect of arthrofibrosis on the medial and lateral gutters, which restrict the internal tibial rotation with knee flexion. Because of the high congruency of the tibiofemoral and patellofemoral joint, an alteration in knee kinematics would lead to persistent knee symptoms in individuals with arthrofibrosis after ACL reconstruction [20]. In clinical practice, restoring normal knee kinematics may be a future therapeutic target in patients with arthrofibrosis after ACL reconstruction.

In our study, we found a significant gender difference in the tibial mediolateral translation and rotation relative to the femur. In female patients, the medial tibial translation was significantly decreased at 60° and 75° knee flexion compared to males. However, an in vitro study showed that in a squatting task females exhibited similar tibial mediolateral movement compared with males [21]. Kartik et al. [22] found no significant gender difference in mediolateral tibial translation in healthy participants. The differences between findings of our study and other studies may indicate changed tibiofemoral kinematics at the arthrofibrotic knee compared to healthy group. Compared to males, female patients showed lower internal tibial rotation at 60° and 75° flexion. These data are consistent with the reported smaller internal tibial rotation in females [22, 23]. From a biomechanical point of view, decreased internal rotation is associated with an increase in the effective Q angle under weight-bearing conditions [24]. This may lead to lateral shift of contact pressure in the patellofemoral joint [25]. This explanation and its relationship to the higher incidence of patellofemoral problems in female patients still need to be studied [26]. The decreased tibial mediolateral translation and rotation during dynamic knee flexion may predispose the female patient to increase the risk of developing knee arthrofibrosis. As such, the findings provide new biomechanical information important to decreasing knee arthrofibrosis and improving clinical outcomes related to patellofemoral problems after ACL reconstruction.

Our study showed that gender had significant effect on inferior patellar shift, which increased with knee flexion in a similar manner in both female and male patients. In the present study, female patients had smaller inferior patellar shift at 15° and 30° of knee flexion compared to males. Previous studies have demonstrated that patellar shift and tilt are affected by changes in tibial rotation throughout the action of the patellar tendon [27]. This study showed that female and male patients had different tibial rotation, so we speculated that gender differences of patellar tracking patterns may attribute to decreased tibial movement in female patients. Significant gender differences were also seen in patellar tilt. In female patients, patella tilted less laterally than in males at 60° and 75° knee flexion. Starting from 15° knee flexion, the patella tilted medially till 30° flexion and thereafter showed consistent lateral tilt in both females and males. This trend is consistent with reports of previous studies on patellar tracking [26, 28]. From 0° to 30° knee flexion the patella does not engage in the femoral trochlea, and beyond 30° flexion, the patella is engaged in the groove and its lateral motion is controlled by the trochlea groove [29]. Arthrofibrosis involving the patellofemoral compartment may change the geometry of the groove, resulting in decreased lateral patellar tilt in female patients. This potentially highlights different kinematic treatment targets between males and females. A better understanding of gender differences in the 6 DOF tibiofemoral and patellofemoral kinematics is important for the diagnosis and treatment of knee arthrofibrosis.

ACL reconstruction is performed to allow patients to return to sport with normal knee joint function that does not lead to loss of motion or radiographic evidence of knee OA in the long term [12]. A number of studies have reported the prevalence of knee arthrofibrosis following ACL reconstruction [2, 5]. Specifically, females are at a higher risk for knee arthrofibrosis and OA after ACL reconstruction than males [3]. Based on current evidence, we propose that gender-based differences in knee kinematics could explain, in part, the higher prevalence of knee arthrofibrosis in females after ACL reconstruction, which requires further exploration. It is possible that the development of knee OA may result from the combined changes in lots of biomechanical variables. Increased knee adduction moment has been reported to be related to the progression of knee OA in patients with ACL reconstruction [12]. Asaeda et al. [13] have identified gender-specific differences in the recovery of knee kinematics during gait after ACL reconstruction, and proposed that biomechanical effects of ACL reconstruction should be separately evaluated for males and females. Sex and the use of a patellar tendon autograft were identified as risk factors of arthrofibrosis in the previous literature [7]. As such, the use of an allograft may be associated with a reduced risk of arthrofibrosis in females, and this warrants further investigations. It is equally important to dissipate inflammation and swelling, and to optimize knee motion before surgery in females [8]. Abnormal knee kinematics may impede the recovery of knee function and increase the likelihood of knee OA, and kinematic testing provides a sensitive tool for early identification of changes in tibiofemoral kinematics and patellar tracking in patients with arthrofibrosis after ACL reconstruction. The gender difference in knee kinematics during weight-bearing activities should be considered when evaluating response to treatment programs after reconstruction.

The limitations of the present study need to be acknowledged in the interpretation of the results. In this study, we do not know if the included patients were loading the arthrofibrotic knee and contralateral knee similarly, and that could be the cause for changes in knee kinematics. Future studies should add a force plate under the foot of the knee being imaged. Kinematic parameters were measured from 15° to 75° knee flexion in arthrofibrotic knees of females/males. Another limitation of this study is that we considered only one weight-bearing activity, the lunge task. Other activities, such as stair climbing and walking, should be investigated in future studies.

## Conclusion

In conclusion, we used combined CT and DFIS to quantify the tibiofemoral kinematics and patellar tracking in female and male patients with arthrofibrosis after ACL reconstruction during weight-bearing knee flexion. Our results showed that in arthrofibrotic knees the range of tibial rotation and patellar motion were significantly decreased compared to the contralateral knee. Our study found lower medial tibial translation and internal tibial rotation in female knees. A significant effect of gender on patellar tracking was also noted. Lower inferior patellar shift and lateral patellar tilt were demonstrated in female knees. These data may have important implications for design of gender-specific surgical treatments and rehabilitation programs for females. These findings warrant further investigations to understand implications of these data for surgical treatments, and to determine factors underlying increased risk of knee arthrofibrosis in females.

## Data Availability

The datasets used and/or analyzed during the current study are available from the corresponding author on reasonable request.

## Abbreviations

ACL: Anterior cruciate ligament
OA: Osteoarthritis
ROM: Range of motion
6 DOF: Six degrees-of-freedom
CT: Computed tomography
DFIS: Dual fluoroscopic imaging system
3D: 3-Dimensional
TEA: Transepicondylar axis
ML: Medial–lateral
AP: Anterior–posterior
SI: Superior-inferior
